# Making Medical Education Courses Visible: Theory-Based Development of a National Database

**DOI:** 10.2196/62838

**Published:** 2025-04-16

**Authors:** Andi Gashi, Monika Brodmann Maeder, Eva K. Hennel

**Affiliations:** 1 Medizinische Fakultät, University of Bern, Bern, Switzerland; 2 Forschung und Entwicklung, Schweizerisches Institut für ärztliche Weiter- und Fortbildung SIWF/ISFM, Elfenstrasse 18, Bern, 3006, Switzerland, 41 315030600; 3 Universitätsklinik für Notfallmedizin, Inselspital Bern, Universitätsspital Bern, University of Bern, Bern, Switzerland

**Keywords:** curriculum mapping, faculty development, competencies, database, medical education

## Abstract

**Background:**

Medical education has undergone professionalization during the last decades, and internationally, educators are trained in specific medical education courses also known as “train the trainer” courses. As these courses have developed organically based on local needs, the lack of a general structure and terminology can confuse and hinder educators’ information and development. The first aim of this study was to conduct a national search, analyze the findings, and provide a presentation of medical education courses based on international theoretical frameworks to support Swiss course providers and educators searching for courses. The second aim was to provide a blueprint for such a procedure to be used by the international audience.

**Objective:**

In this study, we devised a scholarly approach to sorting and presenting medical education courses to make their content accessible to medical educators. This approach is presented in detailed steps and our openly available exemplary database to make it serve as a blueprint for other settings.

**Methods:**

Following our constructivist paradigm, we examined content from medical education courses using a theory-informed inductive data approach. Switzerland served as an example, covering 4 languages and different approaches to medical education. Data were gathered through an online search and a nationwide survey with course providers. The acquired data and a concurrently developed keyword system to standardize course terminology are presented using Obsidian, a software that shows data networks.

**Results:**

Our iterative search included several strategies (web search, survey, provider enquiry, and snowballing) and yielded 69 courses in 4 languages, with varying terminology, target audiences, and providers. The database of courses is interactive and openly accessible. An open-access template database structure is also available.

**Conclusions:**

This study proposes a novel method for sorting and visualizing medical education courses and the competencies they cover to provide an easy-to-use database, helping medical educators’ practical and scholarly development. Notably, our analysis identified a specific emphasis on undergraduate teaching settings, potentially indicating a gap in postgraduate educational offerings. This aspect could be pivotal for future curriculum development and resource allocation. Our method might guide other countries and health care professions, offering a straightforward means of cataloging and making information about medical education courses widely available and promotable.

## Introduction

Ensuring high-quality health care necessitates the presence of well-trained medical educators [1]. Internationally, this has led to the development of frameworks that define their role and the creation of educational pathways for certification in many predominantly Anglophone countries. The terms “medical education” and “medical educator” are used with varying definitions in various settings. For this study, we define “medical educators” as the diverse group of health care professionals who teach, but regarding their learners, we focused on undergraduate medical students and physicians only. Hence, we include medical educators who are active in multiple settings: from clinical supervision and classroom instruction to the development and implementation of curricula. Their roles span the continuum of medical education from undergraduate through postgraduate training and continuing professional development.

Recent studies have identified significant tensions faced by medical educators, including lack of defined career structures, insufficient recognition of teaching roles, and challenges in developing educational identities [2,3]. While formal training programs alone cannot fully address these structural issues, they serve as a crucial stepping stone in professionalizing medical education.

Globally, this coincides with the effort to provide these educators with a defined career path and support for their teaching activities. Notably, the Royal College of Physicians and Surgeons in Canada has introduced a “Clinician Educator Diploma,” rooted in the findings of studies by Sherbino et al [4,5]. Similarly, the University of Michigan Medical School in the United States offers a Master of Health Professions Education [6-8], and in the United Kingdom, a variety of programs align with the “Professional Standards for medical, dental, and veterinary educators” established by the Academy of Medical Educators [9]. These initiatives exemplify the efforts to emphasize the value of quality medical education and facilitate access to quality training for educators.

Drawing on Wenger’s Communities of Practice theory [10], these structured pathways and frameworks serve not only as career development tools but also as boundary objects that connect different medical education communities and facilitate knowledge sharing. When educators can easily identify and access training opportunities, they can more effectively participate in and contribute to their professional community. The visibility and accessibility of educational resources play a crucial role in fostering community development and knowledge exchange across different regions and language groups, ultimately supporting the growth of medical education as a professional field.

### Lack of Career Pathways and an Opaque Landscape

Nevertheless, many regions internationally do not provide a career pathway for medical educators, nor do they use specific frameworks yet [3,11,12]. While some countries offer incentives such as continuous medical education (CME) credits for educational training, this is not standardized. Similarly in Switzerland, formal requirements for teaching qualifications are not yet standardized; there are some existing incentives for medical educators to pursue didactic training. Many didactic courses, particularly those offered or recognized by the Swiss Institute for Postgraduate Medical Education (SIWF), provide CME credits, independent from whether they concern undergraduate, postgraduate, or CME. Additionally, some specialty training programs, such as Internal Medicine, have incorporated mandatory didactic courses into their curriculum requirements [13]. However, these incentives remain fragmented and vary across specialties and institutions.

Still, in everyday reality, senior staff members are expected to educate while juggling everyday clinical tasks with providing education for students and junior doctors, often lacking dedicated time for teaching activities, as described in literature from Australia, Canada, and the United Kingdom [14-17] and in a 2008 Association for Medical Education in Europe guide [18]. Similarly, in Switzerland, where this study took place, there are few examples of clinics hiring specific teaching staff or at least dedicating specific hours in employment contracts to teaching. This practice is still not the norm and is rarely seen outside of specific pilot programs [19]. All of this leads to a diverse, somewhat undefined, and opaque medical education landscape and hinders high-quality medical education.

### Aims and Research Questions

The aims of this study are twofold. The first aim of this study was to conduct a national search, analyze the findings, and provide a presentation of medical education courses based on international theoretical frameworks to support mainly Swiss course providers and educators searching for courses.

The second aim was to provide a blueprint for such a procedure to be used by the international audience.

Our research questions are (1) How do we conduct the search for courses and their content? and (2) How can we present the courses in an accessible way that is translatable to other regions?

## Methods

### Research Paradigm and Use of Theory

Guided by a constructivist research paradigm, which implies that no objective reality exists but that knowledge is created by social interactions [20], this study adopted a subjectivist inductive approach, using a theory-informed inductive data analysis method, described by Varpio et al [21].

This approach allowed a dynamic interplay between intermediate results and potential theoretical frameworks during data collection until we found the most fitting theoretical lens. This reflective and adaptive approach also ensured the relevance of the study results to the roles and competencies of medical educators as they were reflected in the Swiss data.

After a literature review of possible frameworks [4,5,7,9,22-29] and thorough deliberation by the research team, we selected the framework by Sidhu et al [28] as the best fit to support data analysis. Our decision to adopt this framework was strategic, not only for its clear enough lens through which to categorize and analyze course content, due to its comprehensive integration of 67 texts on educator competency domains, but also because it encompassed multiple health care professions, not just physicians. It comprehensively synthesizes educator competencies and identifies 6 distinct domains: “Teaching and Facilitating Learning,” “Designing and Planning Learning,” “Assessment of Learning,” “Educational Research and Scholarship,” “Educational Leadership and Management,” and “Educational Environment, Quality, and Safety.” This approach provides a robust, inclusive structure for understanding and evaluating educator competencies across different health professions.

This study lays the groundwork for a national strategy in Switzerland to enhance the quality of medical education. The first step was establishing a comprehensive database of existing educational offerings. This database facilitates an iterative and reciprocal examination of the course landscape, identifying gaps in the current educational landscape and recognizing requirements that may diverge from international frameworks. This approach ensures that the subsequent framework development and the certification of educators and courses are tailored to the unique needs and priorities of the Swiss medical education system. Additionally, it also allows us to identify and highlight areas lacking coverage by comparing with existing provisions.

### Selection and Eligibility Criteria for Course Inclusion and Exclusion

Our study employed a systematic approach to searching for courses, with carefully defined inclusion and exclusion criteria.

The inclusion criteria encompassed the geographical scope, that is courses offered in Switzerland or online courses offered by Swiss institutions; the target group that is courses aimed at educators teaching physicians or medical students; and the content focus, that is courses with a primary focus on teaching skills.

The study’s exclusion criterion was individual (1-to-1) offerings.

The limitation of the geographical scope ensured feasibility and was chosen with regard to the first study aim of building a national database. Our target group of educators who teach medical students and physicians, rather than including educators for learners in all health care professions, was driven by several methodological and practical considerations. First, our research team’s expertise lies specifically in physician education, allowing us to conduct more nuanced and informed analysis within this domain. Second, our primary data collection method through the Joint Commission of Swiss Medical Schools (SMIFK/CIMS) naturally oriented our study toward medical education targeting physician settings.

With the formulation of the content focus in teaching skills, we deliberately excluded courses with generalized skills that could apply to any professional setting. These "soft skills" courses—such as generic presentation techniques, leadership pitching, or broadly applicable communication strategies—were set aside to concentrate on educational content specifically tailored to medical teaching contexts.

These criteria helped to refine the search and survey strategies, allowing for a targeted collection of data that is pertinent to the goals of this study.

### Data Collection

#### First Phase: Online Search

We initiated our investigation with an extensive online search (May to July 2023), employing various search strategies to explore clinic, faculty, university, and specialists’ association websites to find information on courses. We approached the search from an estimated end-user perspective, simulating how a medical professional seeking to expand their teaching skills might investigate educational opportunities. This meant using primarily search engines and direct institutional websites (including every medical faculty n=11, university hospital n=9, cantonal and larger regional hospital websites n=18, medical associations n=45, and the official SIWF website). Our search extended across Switzerland’s linguistic diversity, using keywords in the country’s 3 official languages (German, French, and Italian) and English to ensure no regional offerings were overlooked. Exemplary search strings, detailed in Multimedia Appendix 1, were designed to capture the multifaceted nature of medical education across different linguistic and regional contexts. Additionally, we used a pragmatic snowball-like method where we let discovered sources lead to additional course offerings. While not claiming absolute comprehensiveness, this method aimed to provide an overview of medical education courses in the Swiss context.

#### Second Phase: Widening the Search

Subsequently (July to December 2023), we sent out an e-mail targeting representatives within the Joint Commission of the Swiss Medical Schools (SMIFK/CIMS), a commission that includes deans and other stakeholders from all Swiss medical faculties. (see Multimedia Appendix 1 for survey questions). The survey did not systematically track response rates or potential selection biases. To widen our scope and better represent non-university medical institutions, we distributed a modified version of the survey to Human Resources departments of 25 larger hospitals in Switzerland in December 2023.

Through this dual approach of detailed online searches and surveys, we sought to capture a comprehensive snapshot of medical education offerings in Switzerland. It allowed us to gather insights not only from academic institutions but also from larger health care providers, thus offering a comprehensive view of the medical education landscape in Switzerland.

Course data were collected using a spreadsheet attached to the surveys, asking the respondents to fill in information about their courses and refer us to additional information if applicable. The specific metadata sought is presented in Table 1. Missing data were filled out by the authors, either by referring to online information or by consulting with the respective course contact persons directly. This step was important to enrich and verify the information found via the online search.

**Table 1.** Course metadata Items.

Item	Description
Title	Course title
Course ID	ID number applied to the course by the researchers
Provider	Institution providing the course
Contact information	Contact information for further inquiries (contact person and, if available, e-mail)
URL	Link to the course website (if available)
Location	Onsite or blended or hybrid or online
Duration	Duration of course as specified by the provider
Language	Course language (German, French, Italian, and English)
Costs	Costs of the course in Swiss Francs (CHF)
Certification	If the course yields a certification of attendance, title, or similar
Accreditation	Number of CME (continuous medical education)—credits or ECTS—credits for a study program
Target audience	For example, Attending Physicians in student care, all health care staff, bedside tutors, etc

### Data Analysis

In the data collection phase, we observed an interplay between the course offerings and our theoretical framework, Sidhu et al [28]. It is important to note, however, that despite our openness to various health care professions at the start of the project, we ultimately focused our analysis exclusively on courses aimed at educators teaching physicians or medical students. This decision was made to maintain the scope of our study within manageable bounds, ensuring our research remained focused and relevant to our primary audience.

Our intention to mirror the actual landscape of Swiss medical education in its present state guided the database construction. Therefore, the selection of keywords for sorting courses and the strategy for their visualization were meticulously developed to emphasize prevalent topics and reveal the intricacies of course content, ensuring our methodology resonates with the real-world context of medical education. We also aimed to maintain the original terminology and language of course descriptions where possible to respect Switzerland’s multilingual landscape and to accurately reflect the providers’ offerings.

### Building the Course Database

We chose Obsidian [30] as our platform for several practical reasons: its intuitive interface and its free access model for educational use made it accessible for users without extensive technical expertise, aligning with our goal for easy usability and adaptability. Obsidian allows for direct, interactive engagement with the data; this feature not only facilitated a more nuanced understanding of the data but also enabled us to refine and validate our keyword mapping and course organization strategies. Furthermore, Obsidian offered an integrated solution for both analyzing and publishing the data online, using Obsidian Publish [30], simplifying the transition from data collection to dissemination. This integration was particularly advantageous for providing an interactive, accessible resource for medical educators and ensuring the database’s utility extended beyond our research team.

The spreadsheet data were then transferred by hand into an Obsidian database, with each course receiving its own Markdown file. Each file included a metadata section on the course and expanded sections on course descriptions, learning objectives and target audience, if available. All course data is provided in the original language and, if not already in English, was translated into English using DeepL, a free online translation tool [31].

### Course Keywording, Standardization, and Refinement of Terminology

**Figure 1.**
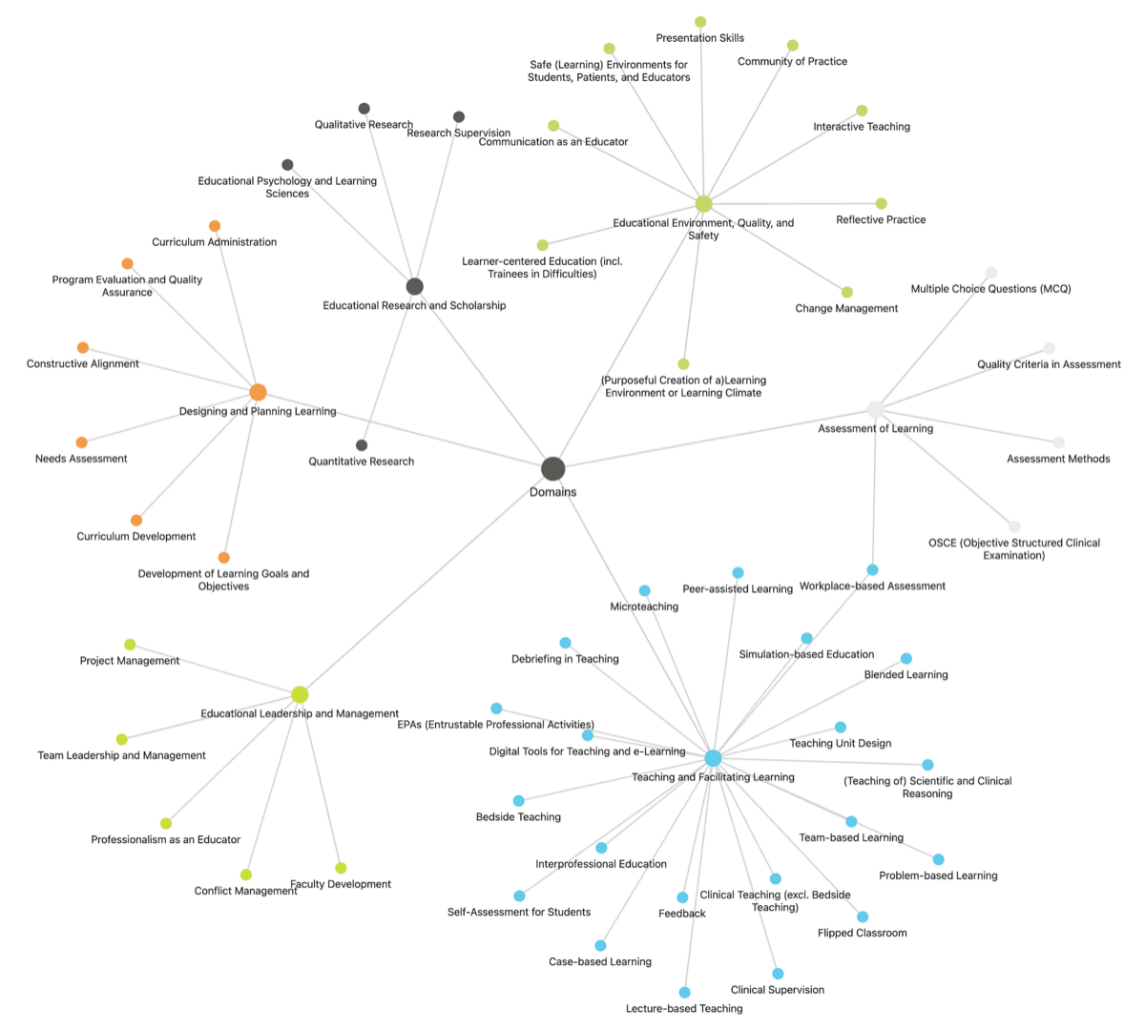
Obsidian visualization of domains and keywords.

AG initially reviewed the compiled comprehensive course data to refine and validate our approach to organizing Swiss medical education course data, identifying preliminary keywords reflective of the content’s breadth. Subsequently, AG and EH collaborated to align these keywords with the competency domains and definitions provided by Sidhu et al [28], ensuring a robust mapping grounded in established educational frameworks (see Multimedia Appendix 2 for a visual representation of the mapping process). The interplay between the domains provided by Sidhu et al. and the keywords as displayed in Obsidian.md is visualized in Figure 1.

AG initially reviewed the compiled comprehensive course data to refine and validate our approach to organizing Swiss medical education course data, identifying preliminary keywords reflective of the content’s breadth. Subsequently, AG and EH collaborated to align these keywords with the competency domains and definitions provided by Sidhu et al [28], ensuring a robust mapping grounded in established educational frameworks (see Multimedia Appendix 2 for a visual representation of the mapping process). The interplay between the domains provided by Sidhu et al and the keywords as displayed in Obsidian.md is visualised in Figure 1 .

This thorough process not only enhanced the accessibility and navigability of course data but also underscored our commitment to precision and educational integrity in documenting the landscape of medical education in Switzerland.

In our methodology, we iteratively developed a set of keywords to authentically represent various aspects of Switzerland’s medical education landscape. Each keyword, crucial for clarity and precision, was documented and defined in separate Markdown files within Obsidian. Notably, integrating these definitions directly into Obsidian’s Markdown documents enabled the keywords to serve as interactive reference points. By simply hovering over them, users can access a preview of the definition, improving the coherence and navigability of our documentation.

### Database Publication

In the development of our study, we aimed to create a comprehensive and interactive database encapsulating the entirety of Switzerland’s medical education landscape, specifically designed for ease of use and utility by medical educators. To make Switzerland’s medical education course offerings easily navigable and useful for the academic community, we used Obsidian Publish [30] for the database’s implementation. This platform was selected to ensure that the database was as interactive and accessible as possible.

To strengthen the trustworthiness of our search strategies we directly contacted each course provider to verify the representation of their course content and confirm the currency and correctness of the information.

Additionally, we prepared to release a template on GitHub to ensure our methodology was transparent and could be replicated in other contexts. This template provides the structure and template files necessary for creating a similar database, focusing on the organization and presentation of data. By offering these template files, we intended to facilitate the adoption of our approach by researchers or educators interested in developing educational landscape databases for different settings. The combination of using Obsidian Publish for the database and GitHub for sharing the template underscores our commitment to accessibility, transparency, and the potential for our work to be adapted and applied broadly.

### Reflexivity

Acknowledging the importance of reflexivity for our constructivist approach, we critically examined the influence of our roles and backgrounds on this study. Each researcher brought distinct perspectives to conceptualizing a medical educator’s role: AG started this project shortly after finishing his studies and provided insights on studying medicine, role definitions, and teaching cultures from a German-speaking and Italian-speaking Swiss region. MB, with a robust background as a practicing internal and emergency medicine doctor and a Master of Medical Education from the University of Bern obtained in 2006, brought insights both from her tenure as president of the SIWF and her experience in teaching emergency medicine courses. EH transitioned from clinical practice and medical education in Germany (Master of Medical Education in 2020) into medical education research in Switzerland (PhD in Medical Education), enriching our project with her comparative view and interest in theoretical concepts. To further enhance the validity of our keyword selection and study design, we engaged with 3 medical educators from Germany and Switzerland who have vast experience in curriculum development. Their feedback was instrumental in refining our course keywords, ensuring our framework resonated with the complexities and nuances of medical education across different linguistic and educational landscapes.

### Study Setting

The present study was carried out in Switzerland, with a focus on identifying and analyzing medical education courses available within the country. The data collection phase spanned from May 2023 to December 2023. Subsequent data processing occurred concurrently and extended until March 2024.

### Ethical Considerations

As no participants were involved, no ethical approval was needed. Everybody who supported this study by providing data, did so voluntarily without any incentives or conflicts of interest.

## Results

### Courses and Keywords

**Figure 2.**
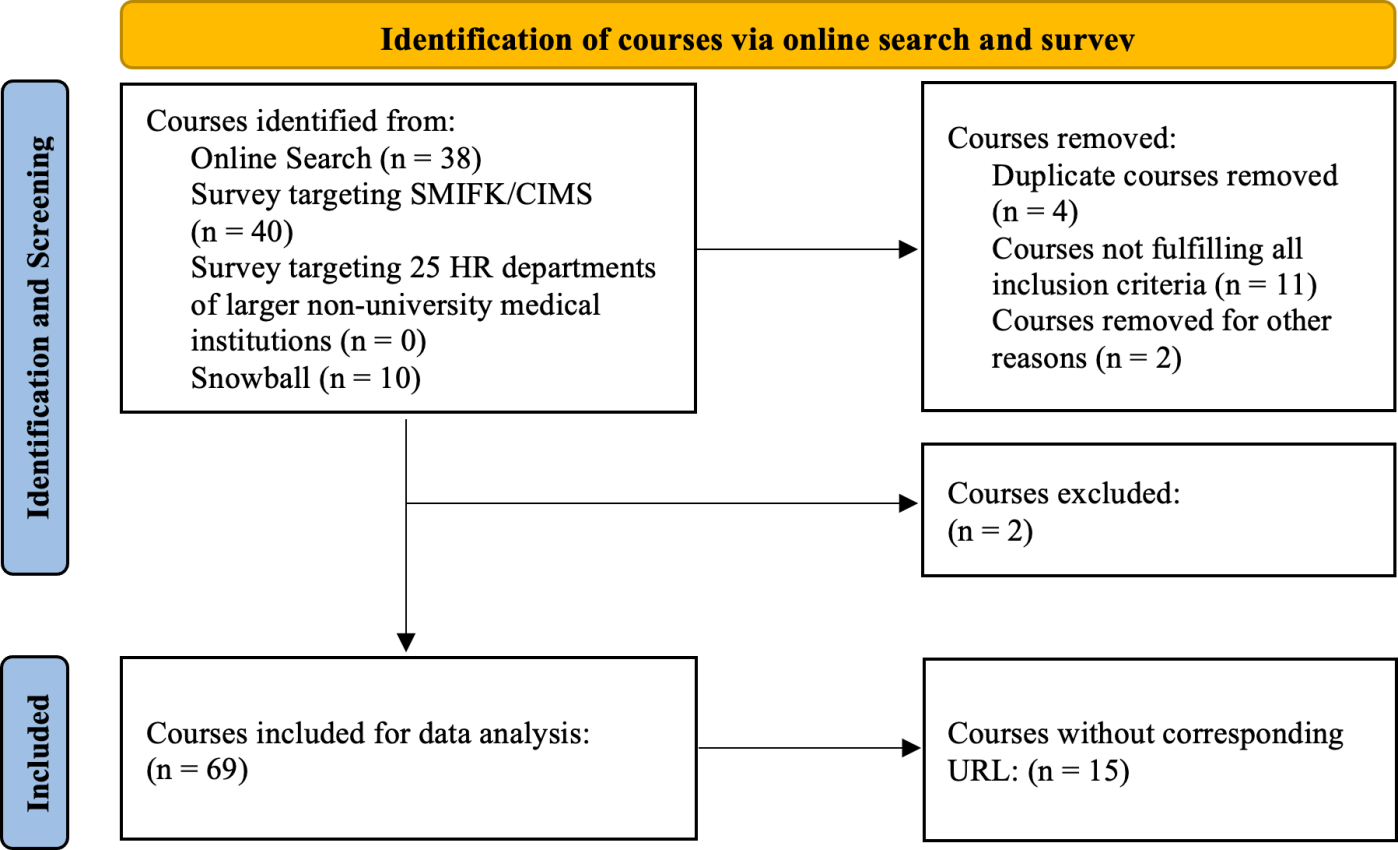
Flowchart of the course identification process. One course that omitted whether physicians were part of its target audience expressed the wish upon further inquiry not to be included in this study for now but to be re-evaluated in future similar endeavors due to the current reorganization of the course offerings. Another course provider told us upon request for further information that our information on their course provided as a survey result was outdated and that they could not share further details about their course. Adapted from Page et al [32].

The initial online search, including the snowball method-like approach, resulted in 38 courses. The survey targeting the SMIFK/CIMS members resulted in 40 additional courses. The survey targeting Human Resources departments of 25 larger, non-university medical institutions in Switzerland yielded no additional new courses. In total, our search method yielded 69 eligible courses, as visible in Figure 2. Of the 69 total eligible courses identified, 36 were found through online searches alone, while 33 were discovered exclusively through survey responses.

To ensure the reliability and currency of our findings, we checked back our data with the respective course providers. Of the 69 courses, 54 had active URLs, while 15 courses were documented without direct web links. These courses were primarily identified through institutional contacts and verified through direct communication in order to maintain the integrity of our comprehensive search strategy.

In our findings, undergraduate teaching courses outnumbered those aimed at postgraduate education (55 out of 69 courses aimed at undergraduate training), while most courses were offered by medical faculties (50 out of 69). The majority of courses were offered in French. In total, 11 courses were offered either additionally or exclusively in English. Most courses were offered onsite, meaning in an offline, face-to-face setting. The descriptive statistics derived from the course metadata, summarizing language use, provider types, course duration, and pricing, are summarized in Table 2.

**Table 2.** Aggregated course statistics.

Description	Count
Total number of courses	69
Average cost (CHF)	880
Courses offered free of charge	28
Courses with unknown costs	10
Course location	
Onsite	50
Hybrid	7
Blended	4
Online	6
Unspecified	2
Course provider	
Swiss Institute for Postgraduate Medical Education (SIWF)	8
Medical faculties	50
Specialists’ associations	3
University hospitals	5
Other	3
Course language^a^	
German	36
French	40
Italian	7
English	11
Romansh	0
Target setting^b^	
Undergraduate teaching	55
Postgraduate teaching	28
Duration, h, average (range)	42 (1.5–8000)

a The count for languages may exceed the total number of courses because some courses are offered in more than one language.

b The count may exceed the total number of courses because some courses target both teaching settings.

In matching the course content across the 6 educational competency domains delineated by Sidhu et al, we identified 52 keywords. The distribution of these keywords was uneven, with 42% (22/52) of the keywords falling into the “Teaching and Facilitating Learning” domain. In contrast, the domains of “Educational Leadership and Management” and “Assessment of Learning” were represented to a lesser extent, with only 5 keywords each, while “Educational Environment, Quality, and Safety” had 10 keywords. The “Educational Research and Scholarship” domain showed the least coverage with 4 keywords. Individual courses varied in their keyword coverage, with an average of 4 keywords per course (median: 3); one comprehensive course covered 33 keywords across domains. The distribution of keywords across domains is presented in Table 3, with a graphical overview represented in Figure 1.

**Table 3.** Domains and keyword distribution among courses.

Keywords	Keywords per domain
Assessment of Learning	5
Designing and Planning Learning	6
Educational Environment, Quality, and Safety	10
Educational Leadership and Management	5
Educational Research and Scholarship	4
Teaching and Facilitating Learning	22
Keywords covered per Course (average, median, max)	4, 3, 33
Sum of keywords	52

### Database and Template Publication

**Figure 3.**
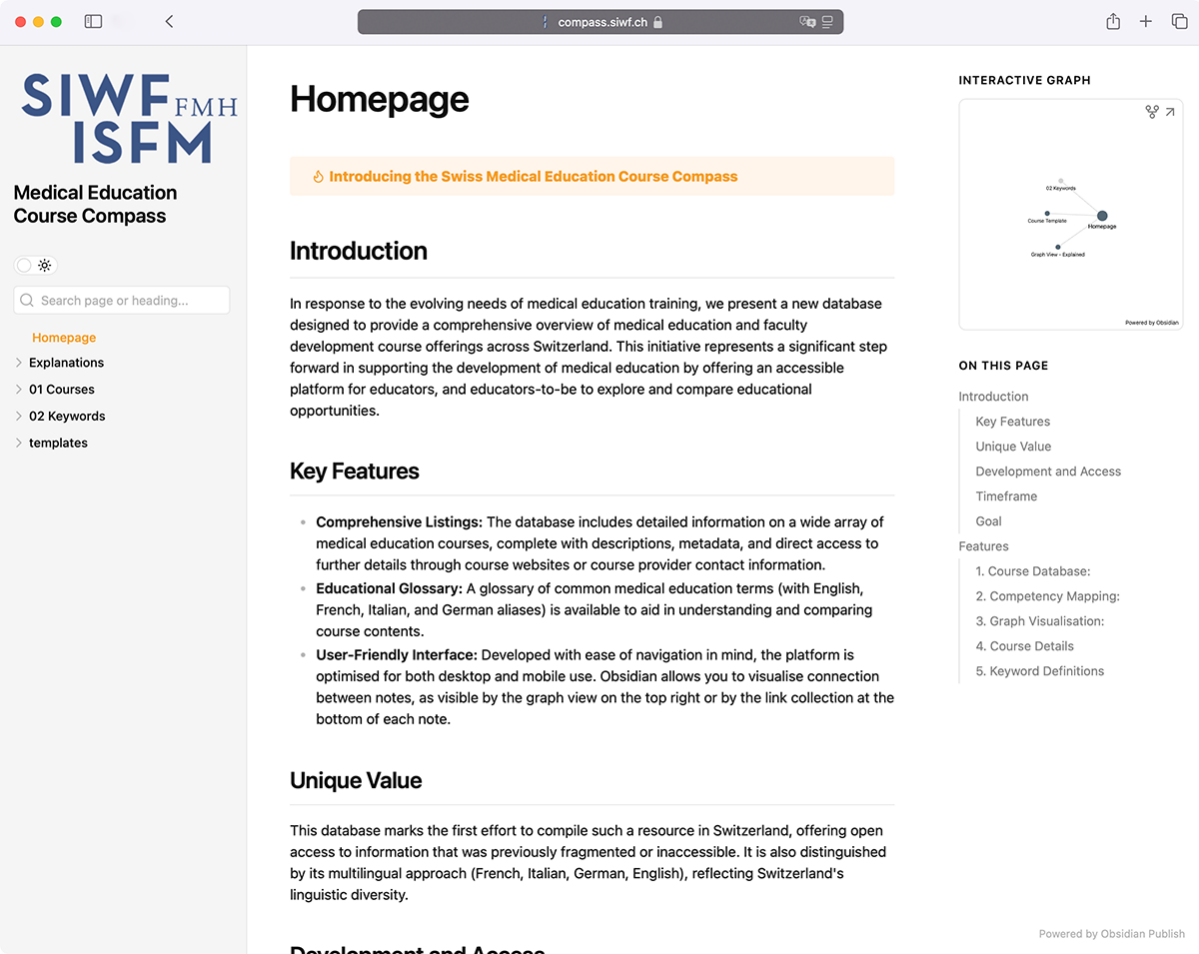
Screenshot of the publicly accessible database landing page.

Using Obsidian publish, the database containing the Swiss Course Data were published online [33] (Screenshots of the landing page and an exemplary course are provided with Figures3  4, please refer to Multimedia Appendix 3 for a video example of navigating the database). We also published an exemplary, ready-to-use database template with a Markdown folder and file structure as a GitHub-Repository, together with instructions explaining the structure and use of the repository to facilitate adoption by other users [34].

**Figure 4.**
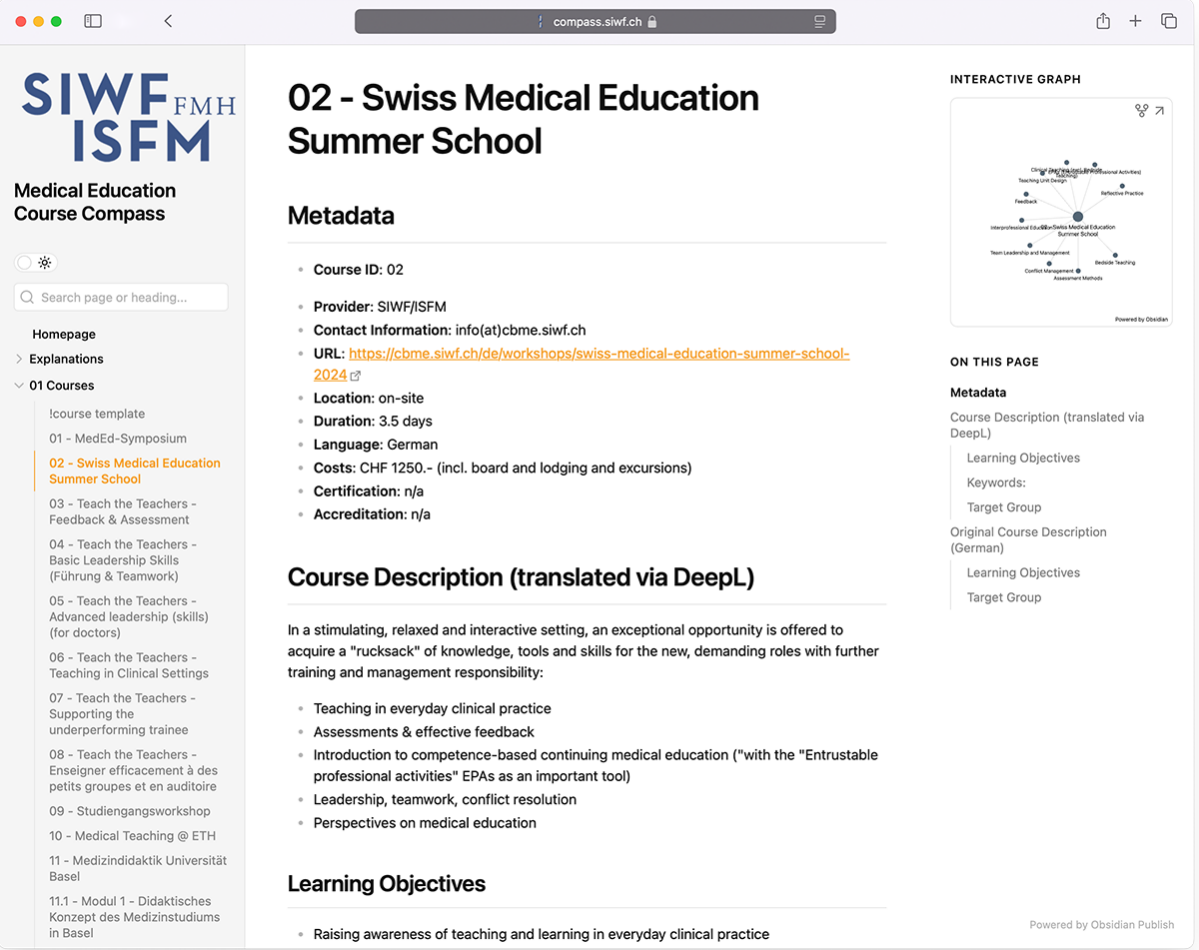
Exemplary screenshot of one course.

## Discussion

### Principal Findings

In line with the 2 aims of this study, our results cover 2 aspects. First, we searched for, analyzed, and presented the Swiss courses. We found that 69 courses are provided, of which 55 target teachers of undergraduate learners and 28 target teachers of postgraduate learners. The courses were offered in several languages (52% German, 58% French, 10% Italian, 16% English) and several formats (72% onsite, 10% in a hybrid manner, 6% as a blended-learning format, and 9% online). Course content covered mainly the “Teaching and Facilitating Learning” domain, which contained 42% (22/52) of all identified keywords and was also the most prominent in course offerings, with the keyword ’feedback’ being featured most frequently, appearing in 40.6% (28/69) of all courses.

Second, we created an internationally transferable strategy based on an international framework, using free-for-educational-use software, with published search strings to be adapted by others, and providing all metadata open access.

This study addressed the need for an approachable method of sorting and displaying courses by providing a comprehensive, accessible database of medical education courses in Switzerland. This initiative is particularly relevant in contexts where medical education training lacks clear structure and uniformity, especially across different linguistic and cultural regions.

While we acknowledge that our search strategy may not have uncovered every course, we believe this data set sufficient for our study’s objectives and that it reflects the real-world challenges faced by aspiring medical educators in navigating the educational landscape.

### Challenges in Navigating the Medical Education System

Several pervasive challenges exist in medical education. These include a discernible lack of dedicated teaching time [35], insufficient, non-transparent, or misdirected funding [36], and an absence of recognition for educators within the medical field [37]. Such issues create a universal backdrop against which specific national education systems can be analyzed.

Our research provides a structured analysis of medical education courses in the Swiss system, illuminating both publicly accessible offerings and those that emerge through professional networks. Significantly, nearly half of the identified courses (33 out of 69) were discovered only through contact with course providers rather than public online searches, revealing a critical accessibility gap. This finding suggests that course discovery often depends heavily on existing professional networks and insider knowledge, potentially creating barriers for newcomers to the field who lack such connections. This study is the first of its kind to investigate medical didactics courses in a structured manner in Switzerland, providing an overview and allowing new perspectives.

Our study uncovers a complex challenge within the Swiss medical education system: navigating the educational landscape shaped by 4 national languages—German, French, Italian, and Romansh. Among these, German, French, and Italian are official languages. In this multilingual setting, English emerges as a crucial lingua franca in international research and academia and as a common medium within Switzerland, facilitating communication across linguistic barriers. This is reflected in the educational offerings, with 11 courses being conducted partially or entirely in English. Such linguistic plurality further aggravates the existing variability in didactic methods and medical education and research terminology [38,39], presenting additional challenges to the creation of an educational framework. This situation is further complicated by issues of information accessibility—with 15 courses not having available URLs, it renders it difficult for potential participants to find accurate, up-to-date information about these educational opportunities online.

### Implications for Medical Educator Career Pathways

Building on the initial findings, our work, as the first comprehensive compilation of available didactic courses within the Swiss medical education system, can potentially enable more transparency in the field. By illuminating the current educational offerings, our research could facilitate faculty development and inform career choices for medical educators, as they now have a more straightforward overview of the resources and opportunities available to them. This transparency is a critical step towards enhancing career pathways, as it allows for a strategic approach to personal and professional development within the context of medical education.

However, it is essential to note that our study revealed a predominance of courses aimed at teaching in undergraduate (medical school) settings. While this finding initially suggests a potential undersupply of educational opportunities targeting postgraduate teaching, it notably reflects Switzerland’s institutional structure, where universities are primarily responsible for undergraduate medical education but not residency training or CME. This structural arrangement, where different institutions oversee different stages of medical education, may contribute to the observed imbalance in course offerings. The relative scarcity of training for postgraduate settings might indicate a need for a broader array of courses catering to the ongoing development of medical professionals beyond their initial degrees. Addressing this gap could lead to a more robust and comprehensive educational framework, ensuring that medical educators are well-equipped to foster the next generation of medical practitioners at all stages of their professional journey.

### Comparison to International Models

Our comparative analysis with international models highlights notable differences in structuring medical education career pathways. Particularly in Anglophone countries, such as Australia, Canada, the United Kingdom, and the United States, well-established career pathways and educator frameworks are actively promoted and used, unlike in Switzerland, where such structured frameworks are absent [3,11,12].

When aligning our findings with the integrative framework by Sidhu et al [28], which outlines 6 educator competency domains, we observed that Swiss medical education predominantly emphasizes the domain of “Teaching and Facilitating Learning.” This domain is focused on enhancing learning through suitable methods and resources, including assessment for learning. This heavy focus may inadvertently lead to a neglect of the other 5 domains (“Educational Leadership and Management,” “Educational Environment, Quality, and Safety”, “Designing and Planning Learning,” “Assessment of Learning,” and “Educational Research and Scholarship”), suggesting a possible imbalance in the educational emphasis. Our keyword map corroborates this emphasis, revealing a detailed and nuanced depiction of the “Teaching and Facilitating Learning” domain. Our mapping strategy aimed to reflect the specificity and depth of course offerings without excessive summarization, thus indicating their relative prominence by the frequency with which they appear in our data set.

The predominance of the “Teaching and Facilitating Learning” domain could stem from its direct applicability in educational settings and the relative ease of teaching and assessing these skills. This focus on tangible teaching methods and resources, which offer clear benefits and outcomes, could make it a natural focal point for educators aiming to directly impact student achievement. However, this emphasis may inadvertently lead to the undervaluation of other critical educator competencies, such as “Educational Leadership and Management” or “Educational Research and Scholarship.” These domains encompass more abstract competencies that resemble attitudes or overarching professional dispositions rather than concrete skills, presenting challenges for direct instruction due to their less immediately visible impacts and harder-to-quantify qualities.

The challenge of effectively imparting the more abstract domains within medical education and the observable predilection for addressing more accessible teaching topics can be analogized to the tendency to assess readily quantifiable competencies [40]. This parallel might reflect an educational predilection for what can be straightforwardly taught and measured, perhaps at the expense of more profound, more complex competencies that are less amenable to conventional assessment methodologies. Such a tendency may not fully encapsulate the multifaceted nature of medical educator competencies, underscoring a potential disjunction between educational priorities and the comprehensive skill set required for clinical excellence.

The marked predominance of teaching methods and learning facilitation within the Swiss context, as opposed to a more balanced distribution across the 6 domains, is an intriguing phenomenon. We propose that this area warrants further inquiry. Investigating why there is such a strong focus on these teaching competencies within Switzerland, especially compared to the broader scope seen internationally, could yield insights that inform future developments in medical education. This analysis might ultimately contribute to enhancing educator frameworks and diversifying professional development opportunities.

### Future Directions and Potential Impact

The insights garnered from this study lay the groundwork for developing a unified and expansive framework for medical education in Switzerland, emphasizing the need for a formal recognition and certification process for medical education courses and medical educators. By enhancing awareness of available courses and clarifying the expectations for a medical educator, this initiative could significantly improve the quality of medical education. Increased awareness is crucial for establishing clear competencies in the first place, and it also serves to acknowledge and validate existing efforts while encouraging the development of future educational opportunities. The overview of courses also offers the basis for future discussions, which have already begun. Currently, the database does not give information on the quality of courses, as this could not reliably be assessed. For a future version of the database, methods to assess course quality and display this transparently are developed.

In addition, the overview on courses in comparison with the framework has shown some blind spots of the current training. Courses on “Assessment of Learning,” “Educational Leadership and Management,” and “Educational Research and Scholarship” seem to be rare. As a follow-up to this study, the responsible groups (SMIFK and SIWF) will discuss these gaps and take this as a base for future course offerings.

Furthermore, this focus on a structured framework could resonate on an international level, offering a model for contexts where clear career pathways for medical educators are similarly undefined, thus having the potential to inform and shape international standards in medical education.

While this study focused exclusively on courses on medical education (physician-adjacent) settings to maintain a manageable scope, we deliberately chose Sidhu et al’s framework for its interprofessional applicability across health care education. Combined with our intentionally transparent and replicable methodology, this provides a strong foundation for other health care professions to adapt our approach to mapping their own educational landscapes. The template we developed could serve as a starting point for similar analyses in other health professions’ education systems.

### Limitations

Our study encountered different challenges. The research relied primarily on internet-searchable courses, potentially overlooking non-digital or unadvertised educational offerings. Additionally, our snowball search approaches introduce potential selection biases.

As we focused on assembling an overview instead of the details of courses, the depth of content validation regarding, for example, course quality, pedagogical methodologies, and educational effectiveness is limited.

Future research should focus on course content and course quality and investigate the usability and comprehensiveness of our database, including an accessibility audit of course information, a comprehensive comparison with expert-identified course offerings, and systematic verification of course details. We are planning to investigate the implications of the use of the database, such as changes in content offered and new strategies to acknowledge medical educator careers.

### Conclusions

The methodology showcased in our study serves as a valuable template for international adaptation, offering insights into how diverse educational systems can be evaluated, organized, and presented effectively. By innovatively mapping the educational landscape with the example of Switzerland, we have provided a replicable approach that could aid other countries, even with similar linguistic and cultural challenges. The creation of our database amplifies the visibility and transparency of medical education courses available, facilitating better decision-making for educators. This enhanced access to information allows medical professionals to chart their career progressions more effectively, emphasizing the benefits of more transparent navigation, especially in environments without well-defined educational pathways.

Through this pioneering work, we aim to contribute to the global conversation on medical education, fostering a more interconnected and accessible educational landscape that benefits educators and students alike.

## Supplementary material

10.2196/62838 Multimedia Appendix 1 Document outlining our Search Strategy for the online search.

10.2196/62838 Multimedia Appendix 2 Screenshot of Miro Board used for mapping between domains and keywords which helped facilitate discussions between authors.

10.2196/62838 Multimedia Appendix 3 Video example of navigating the publicly accessible online database

## Abbreviations

CME: continuous medical education
SIWF: Swiss Institute for Postgraduate Medical Education
SMIFK/CIMS: Joint Commission of the Swiss Medical Schools
