# Supplementary figures and images for: Making Medical Education Courses Visible: Theory-Based Development of a National Database
Source: JMIR Med Educ. 2025 Apr 16;11:e62838. doi: 10.2196/62838 (PMC12017612; doi:10.2196/62838)

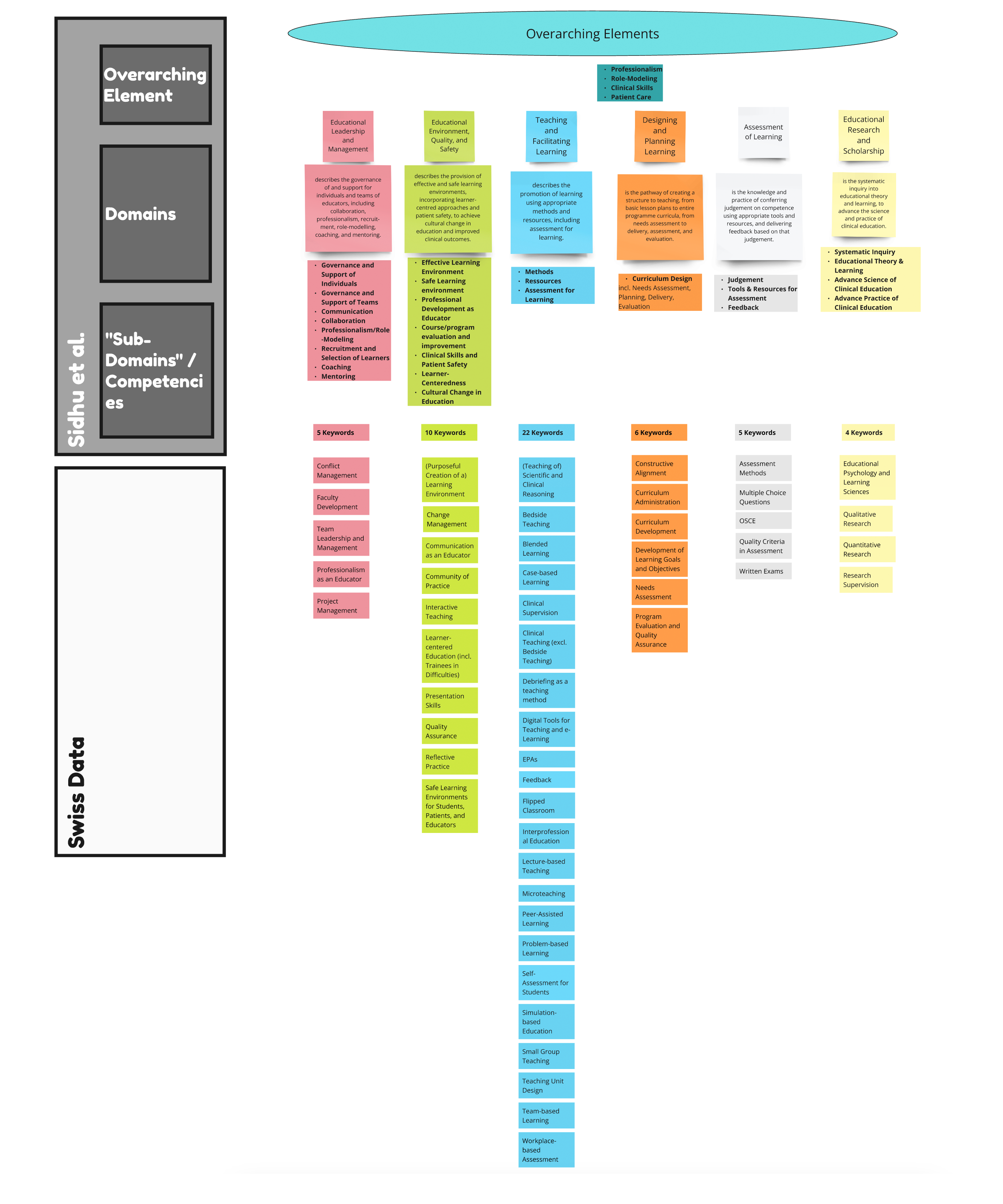

Supplement: Multimedia Appendix 2 [file mededu-v11-e62838-s002.png]
